# A comparison of perinatal outcomes following fresh blastocyst or cleavage stage embryo transfer in singletons and twins and between singleton siblings

**DOI:** 10.1093/hropen/hoad003

**Published:** 2023-03-08

**Authors:** Edwin-Amalraj Raja, Siladitya Bhattacharya, Abha Maheshwari, David J McLernon

**Affiliations:** Institute of Applied Health Sciences, Polwarth Building, University of Aberdeen, Aberdeen, UK; Aberdeen Fertility Centre, NHS Grampian, Aberdeen, UK; Institute of Applied Health Sciences, Polwarth Building, University of Aberdeen, Aberdeen, UK

**Keywords:** blastocyst, cleavage, embryo transfer, perinatal outcome, preterm birth, birthweight, congenital anomaly

## Abstract

**STUDY QUESTION:**

Are perinatal outcomes following fresh blastocyst versus fresh cleavage stage embryo transfer (ET) different in singletons, twins, and between singleton siblings?

**SUMMARY ANSWER:**

Singleton babies conceived following fresh blastocyst, versus cleavage stage, ET are less likely to be small for gestational age (SGA) or to have a congenital anomaly (a result confirmed by comparing singleton siblings), while singletons born following fresh blastocyst ET were at a higher risk of being large for gestational age (LGA) than their sibling born following fresh cleavage stage ET.

**WHAT IS KNOWN ALREADY:**

Blastocyst stage transfer is now the preferred strategy in most IVF units. Previous studies have suggested that babies conceived through blastocyst transfer are at increased risk of preterm birth and LGA.

**STUDY DESIGN, SIZE, DURATION:**

A national population-based retrospective cohort study was performed using linked Human Fertilisation and Embryology Authority (HFEA) data on 130 516 IVF and ICSI livebirths occurring from 103 062 women between 2000 and 2017.

**PARTICIPANTS/MATERIALS, SETTING, METHODS:**

We included women who had at least one singleton livebirth resulting from IVF/ICSI fresh embryo treatment, using their own eggs and partner’s sperm. A linked HFEA dataset was analysed using a multilevel framework, which accommodated repeated IVF cycles resulting in livebirths in the same woman. A population-averaged robust Poisson model was used for binary outcomes and a multinomial logistic regression model was used for categorical outcomes. Unadjusted and adjusted risk ratios (aRRs) (95% CI) were calculated.

**MAIN RESULTS AND THE ROLE OF CHANCE:**

There were 130 516 livebirths in 103 062 women, including 86 630 singletons, 43 886 twin births, and 5384 pairs of singleton siblings. In comparison with fresh cleavage stage ET, fresh blastocyst stage transfer in singletons was associated with a lower risk of low birthweight (aRR = 0.92; 95% CI 0.86, 0.99), lower risk of being SGA (0.83; 0.78, 0.89), and lower risk of congenital anomaly (0.79; 0.71, 0.89). This analysis did not show an increase in risk associated with preterm birth (1.00; 0.94, 1.06), high birthweight (0.99; 0.93, 1.06), LGA (0.99; 0.93, 1.05), and the chance of healthy singleton baby (1.00; 1.00, 1.02). Twins resulting from fresh blastocyst stage ET were at slightly higher risk of preterm birth (1.05; 1.02, 1.10) compared with twins conceived following fresh cleavage stage ET. There was insufficient evidence for an association with the other perinatal outcomes. Singleton siblings born following fresh blastocyst stage ET were at a higher risk of being LGA (1.57; 1.01, 2.46) and at lower risk of having a congenital anomaly (0.52; 0.28, 0.97) compared to their singleton siblings born following cleavage stage ET. There was some evidence of excess risk of preterm birth (1.42; 0.97, 2.23) associated with blastocyst stage transfer. However, we could not confirm an association between blastocyst stage ET and low birthweight (1.35; 0.81, 2.27), high birthweight (1.19; 0.80, 1.77), and the chance of being a healthy baby (0.97; 0.86, 1.09).

**LIMITATIONS, REASONS FOR CAUTION:**

This was an observational study where we were unable to adjust for some key confounders, such as maternal smoking status and BMI, which may change from one pregnancy to another and are not recorded in the HFEA dataset.

**WIDER IMPLICATIONS OF THE FINDINGS:**

In the largest study of its kind, our analysis of singleton siblings, corrected for unmeasured, non-time varying maternal factors, confirms the previously reported association between blastocyst transfer and LGA babies, and shows a reduced risk of congenital anomaly following blastocyst transfer. Our sibling analysis did not confirm a decreased risk of low birthweight following blastocyst transfer. Overall, absolute risks are low and there is insufficient evidence to challenge the practice of extended culture of embryos.

**STUDY FUNDING/COMPETING INTEREST(S):**

This project is financed by an NHS Grampian Endowment Research Grant, project number 17/052. One of the authors, S.B., was the Editor in Chief of *HROpen* until 31 December 2022 and would have been in that role when the paper was first submitted. As an invited speaker, S.B. has received travel expenses, accommodation and honoraria from Merck, Organon, and Ferring. A.M. has received travel expenses, accommodation, and honoraria from Merck Serono, Cook Medical, Pharmasure, Gedeon Richter, and Ferring. D.J.M. is currently a *HROpen* Associate Editor.

**TRIAL REGISTRATION NUMBER:**

N/A.

WHAT DOES THIS MEAN FOR PATIENTS?

IVF embryos are grown in the laboratory for between 2 and 5 days before they are replaced within the womb. The day on which an embryo is transferred (Day 2/Day 3 or Day 5) could affect a pregnancy resulting from treatment and the health of a baby. The aim of our study was to use information from British IVF clinics to find out whether the time an embryo is grown in the laboratory affects its chances of developing into a healthy baby (measured as preterm, low or high birthweight, or congenital anomaly).

Our results show that single babies born following the transfer of embryos on the fifth day are larger than their siblings (brothers or sisters) created using embryos replaced sooner (at Day 3) and have a lower risk of birth defects. Twins conceived following a Day 5 embryo transfer were marginally more likely to be preterm than those born following a Day 3 embryo transfer.

## Introduction

Since the first report of successful blastocyst stage embryo transfer (ET) in 1985 (Cohen *et al.*, 1985), this practice has been widely adopted in many countries (Farquhar *et al.*, 2010; Dar *et al.*, 2014; Kissin *et al.*, 2015; HFEA, 2016; Holden *et al.*, 2018; Marconi *et al.*, 2019; Spangmose *et al.*, 2020). In comparison to the more traditional practice of cleavage stage transfer at 2–3 days after fertilization, extended culture of embryos to the blastocyst stage, which offers potential advantages in terms of embryo selection and better endometrial–embryo synchrony, has been shown to result in improved live birth rates per transfer (Papanikolaou *et al.*, 2006; Wang and Sun, 2014; Glujoversusky *et al.*, 2016). This has encouraged clinics to adopt a policy of single embryo (blastocyst) transfer without compromising live birth rates, and data from national registries suggest that blastocyst transfers are now the preferred option in most IVF and ICSI treatment cycles (Banker *et al.*, 2021; SART, 2021; HFEA, 2021a).

Although perinatal outcomes following blastocyst transfer are mostly reassuring, some observational studies have suggested an increased risk of preterm birth (Kalra *et al.*, 2012; Dar *et al*., 2013; Chambers *et al.*, 2015; Martins *et al.*, 2016; Wang *et al.*, 2017; Alviggi *et al.*, 2018), low birthweight (Maheshwari *et al.*, 2013), small for gestational age (SGA) babies (Kalra *et al.*, 2012; Maheshwari *et al.*, 2013; Chambers *et al.*, 2015; Martins *et al.*, 2016; Wang *et al.*, 2017; Alviggi *et al.*, 2018), and congenital anomalies (Källén *et al.*, 2010).

It is unclear whether these risks are due to the laboratory processes associated with extended culture *per se* or inherent differences in maternal characteristics, as blastocyst transfer is usually undertaken in women who have a number of good quality embryos and tend to have a better IVF prognosis. Most published studies have reported outcomes for singleton pregnancies based on cycle-level analyses of registry data (Sunkara *et al.*, 2014; Marconi *et al.*, 2019) and have been unable to adjust for multiple cycles within a single woman, or report on outcomes in multiples. Absence of linked registry data has meant that most published studies have been unable to adjust for the clustering of IVF cycles within women or disaggregate the impact of maternal factors from those caused by extended culture by comparing perinatal outcomes in siblings conceived following ET at cleavage or blastocyst stage (Romundstad *et al.*, 2008; Kalra *et al.*, 2012).

In this study, we used linked UK IVF data collected by the Human Fertilisation Embryology Authority (HFEA) to compare perinatal outcomes within singletons and within twins conceived following blastocyst versus cleavage stage ET. This dataset contains cycle identifiers within each woman, which allowed us to identify different treatment cycles within each woman. We were therefore able to compare perinatal outcomes between singleton sibling pairs where one child was conceived from a blastocyst, while the other was conceived following a cleavage stage ET.

## Materials and methods

### Database

The HFEA has been the statutory regulator of assisted conception treatment tasked with collecting data on licenced IVF treatment cycles performed in the UK since 1991 (HFEA, 1990, 2021b). We analysed a version of the HFEA database which links all IVF cycles to individual women. Ethical approvals were obtained to utilize the HFEA dataset from the North of Scotland Research Ethics Committee (Ref: 19-YH-0041), the Confidentiality Advisory Group, and the HFEA Register Research Panel. The data were extracted by HFEA and transferred securely to the Data Management Team, School of Medicine, Medical Sciences and Nutrition, University of Aberdeen.

### Study population

We included women who had at least one singleton live birth resulting from a fresh embryo transferred following IVF (including ICSI) treatment in the UK between 2000 and 2017. Babies born following frozen-thawed ET were excluded from the study since day of ET was not available for frozen cycles in the HFEA dataset. As blastocyst stage transfers were infrequent before 2000, we restricted our sample to women treated between 2000 and 2017. We included live born infants whose gestational age was 22 weeks or more, with a minimum birthweight of 500 g. We excluded still births, births in women under 18 years or over 50 years of age, and those involving oocyte donation, embryo donation, preimplantation genetic testing, or surrogacy. Cycles where more than three embryos were transferred were excluded as many of these resulted in triplet and quadruplet births. Births resulting from ETs on Day 6 were excluded as these only involved frozen embryos.

### Exposure

The exposed cohort comprised women who had a live birth resulting from fresh ET on Day 5 of culture (blastocyst stage). The unexposed cohort comprised women who had a live birth following fresh cleavage stage ET on Day 2 or Day 3 of culture.

### Outcomes

The main outcome measures were gestational age at birth, birthweight, congenital anomaly, and ‘healthy baby’, defined as a baby born at or after 37 weeks of gestation, weighing between 2500 and 4000 g with no evidence of any congenital malformations in each of the singletons and each infant in twins (Wang *et al.*, 2010; Marconi *et al.*, 2019). Gestational age was grouped into three categories: very preterm birth (<32 completed weeks of gestation), preterm birth (<37 completed weeks of gestation including very preterm), and full-term birth (≥37 completed weeks of gestation used as reference). Birthweight at delivery was grouped into three categories: low birthweight (<2500 g), normal birthweight (2500–3999 g used as reference), and high birthweight (≥4000 g). In singletons, birthweight was also categorized into SGA, appropriate for gestational age (AGA), and large for gestational age (LGA) using UK-based centile charts of birthweight for gestational age stratified by infant sex and maternal parity (Bonellie *et al.*, 2008). SGA babies were babies whose birthweights were below the 10th percentile for babies of the same gestational age, and LGA babies were those whose birthweights were above the 90th percentile for babies of the same gestational age. AGA babies were those within the 10th to 90th percentile range and used as reference. A small proportion of infants (n = 64) born at 22, 23, or 44 weeks of gestation and missing baby gender (n = 1583) were excluded from this particular analysis as the birthweight reference table did not contain birthweights for these gestational ages. Twins could not be categorized as SGA or LGA because the twin population-based reference chart of birthweight for gestational age is stratified by infant gender and chorionicity (Briffa *et al.*, 2021). Unfortunately, the HFEA dataset does not contain a variable which would allow us to identify twins who are monochorionic or dichorionic.

### Statistical analysis

Descriptive statistics were calculated for each of the women’s characteristics, split by live birth as a result of either a cleavage stage or blastocyst ET.

#### Singleton live birth

The unit of analysis here was a singleton live birth episode resulting from transfer of a fresh blastocyst or cleavage stage ET. As some women had two or more singleton live birth episodes arising from several ETs within the study period, all analyses were conducted under a multilevel framework, which accommodated repeated cycles resulting in livebirths within the same women. In order to account for the dependency between cycles resulting in live birth within women, a population-averaged model using generalized estimating equations was used to explore associations between the exposure groups (blastocyst versus cleavage stage ET) and perinatal outcomes (Hardin and Hilbe, 2003) and to estimate 95% CI using robust standard errors that allowed for correlation within women (McCullagh and Nelder, 1989). We specified an exchangeable correlation structure, which assumes that the risk of a perinatal adverse event was the same for any live birth within a woman. For the outcomes of preterm birth (preterm birth versus full-term birth), congenital anomaly (yes versus no), and healthy baby status (yes versus no), a robust Poisson regression model was used. For the two birthweight outcome variables (i.e. birthweight coded as low, normal or high, and birthweight adjusted for gestational age coded as SGA, AGA, or LGA), a multinomial logistic regression model was employed since each of these variables had three categories (Chamberlain, 1980; Pforr, 2014). The association between treatment strategy (blastocyst or cleavage stage ET) and very preterm birth (versus full-term birth) was estimated using multinomial logistic regression (where we also included 32–37 weeks gestation as a nuisance outcome category). Crude risk ratios (RRs), adjusted RRs (aRRs), and 95% CI were calculated. The following factors were considered as confounders: maternal age (years), cause of infertility (i.e. tubal disease, ovulatory disorder, male factor, unexplained), previous pregnancy status (yes/no), treatment type or type of insemination (IVF versus ICSI), number of eggs collected, and year of treatment. The covariates considered for adjustment differed for each of the outcomes and are listed in the footnote under each table. Since ET stage could influence birthweight through its effect on gestational age, gestational age can be considered to be a mediator on the causal pathway from cleavage or blastocyst stage ET to birthweight. Therefore, it was excluded to avoid bias since its inclusion does not allow us to estimate the total direct effect of the stage of ET on birthweight (Wilcox *et al.*, 2011). In the same way, the number of embryos transferred was considered as a mediator and was excluded from multivariable analyses. Further, congenital anomalies or the underlying cause of congenital anomalies have been linked with iatrogenic preterm birth owing to early induction of labour (Brown, 2009). In this case, gestational age would be considered a collider rather than a confounder as both ET stage and congenital anomaly can affect gestational age through independent routes. Therefore, gestational age was also excluded from this analysis.

#### Twin livebirths

The first set of live born twins was considered for each woman. A very small number of women had a second set of twins, so these were excluded from the analysis for pragmatic reasons. All analyses were conducted under a multilevel framework, which accommodated for twins within the same woman (Carlin *et al.*, 2005; Chambers *et al.*, 2015). For the binary outcomes of preterm birth, congenital anomaly and healthy baby, a Poisson model was used while for the categorical outcomes, term of birth (very preterm birth, preterm birth and full-term birth) and birthweight, a multinomial logistic regression model was employed.

#### Singleton siblings

We compared singleton sibling pairs in which one sibling resulted from a fresh blastocyst-stage ET, while the other was born following a fresh cleavage-stage ET. A fixed effect (conditional) Poisson regression model for paired data was used to compare binary perinatal outcomes (preterm birth, congenital anomaly, and healthy baby) between singleton siblings from the same woman (Carlin *et al.*, 2005). This conditional approach allowed us to measure the RR of a perinatal outcome for a change in ET stage (blastocyst versus cleavage ET), whilst keeping the uterine environment (i.e. the mother’s cycle invariant characteristics) fixed (Neuhaus, 2006). A fixed effect multinomial logistic regression analysis for paired data was used to compare categorical birthweight outcomes (low birthweight versus normal birthweight, high birthweight versus normal birthweight, SGA versus AGA, and LGA versus AGA) between Sibling 1 and Sibling 2 (Pforr, 2014). Therefore, since some of the maternal factors, measured and unmeasured, remained constant between siblings, any observed association between ET strategy and perinatal outcome was related to the transfer strategy (Henningsen *et al.*, 2011; Seggers *et al.*, 2016). The model was adjusted for characteristics that vary from one cycle to another and differed between Siblings 1 and 2, such as maternal age, order of birth as a proxy for parity, treatment type (IVF versus ICSI), number of eggs collected, and year of treatment.

To determine whether the association between treatment strategy and perinatal outcome differed over time, as a secondary analysis, we included an interaction term between treatment strategy (blastocyst ET versus cleavage stage ET) and two time periods (2000–2008 and 2009–2017). We did this for the singleton and twin analysis.

All analyses were carried out using Stata version 17 MP (StataCorp, College Station, TX, USA). A *P*-value <0.05 was considered to be statistically significant.

## Results

**Figure 1.**
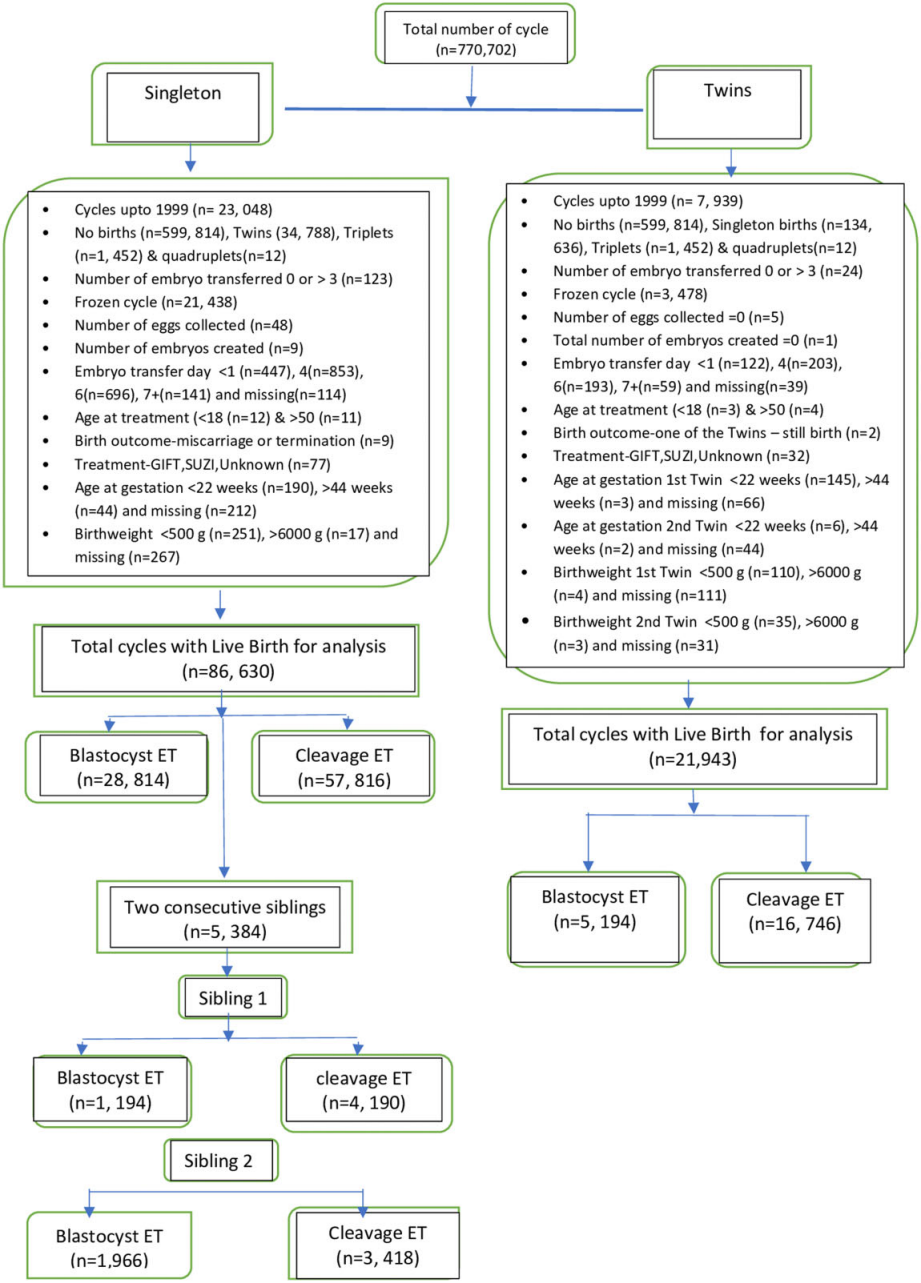
**Flow chart of cohort exclusions in a study of perinatal outcomes.** GIFT: gamete intrafallopian transfer; SUZI: subzonal insemination; ET: embryo transfer.

A total of 130 516 livebirths were included in the analyses (Fig. 1). This included 86 630 singleton livebirths (from 81 119 women), 43 886 twin births, and 5384 pairs of singleton siblings.

### Singleton livebirths

Among singletons, 28 814 livebirths resulted from blastocyst stage transfer and 57 816 from cleavage stage transfer. There were 32 817 (56.7%) Day 2 ETs out of 57 816 cleavage stage ETs. Maternal and treatment characteristics are shown in Table I. On average, women in the blastocyst group were younger, had a higher oocyte yield and were more likely to have had a single ET.

**Table I:** Comparison of baseline maternal and treatment characteristics between women who delivered a singleton live born baby following blastocyst versus cleavage stage embryo transfer.

MATERNAL/COUPLE CHARACTERISTICS	Live births following blastocyst stage ET (N = 28 814) n (%)	Live births following cleavage stage ET (N = 57 816) n (%)	*P*-value
Maternal age at treatment (years), mean (SD)	33.4 (4.1)	33.9 (4.1)	<0.001
Tubal disease	4311 (14.9)	10 954 (18.9)	<0.001
Ovulatory disorder	4544 (15.8)	7081 (12.2)	<0.001
Male factor	11 719 (40.7)	25 908 (44.8)	<0.001
Endometriosis	2007 (6.9)	3917 (6.8)	0.310
Unexplained	8712 (30.2)	15 990 (27.7)	<0.001
Duration of infertility (years), median (IQR)	4 (3, 7)	4 (3, 7)	<0.001
Missing	26 118	25 184	
Previous live birth	2588 (8.9)	5190 (8.9)	<0.001
Type of fertilization			<0.001
IVF	12 981 (45.1)	27 422 (47.4)	
ICSI	15 833 (54.9)	30 394 (52.6)	
Number of eggs collected	12 (9, 16)	9 (6, 13)	<0.001
Number of embryos transferred			<0.001
1	18 841 (65.4)	7372 (12.8)	
2	9670 (33.6)	46 477 (80.4)	
3	303 (1.1)	3967 (6.8)	
Elective single ET			<0.001
Yes	15 722 (54.6)	3132 (5.4)	
No	13 092 (45.4)	54 684 (94.6)	

The associations between baseline maternal and treatment characteristics and blastocyst (versus cleavage stage) embryo transferred were examined using a Poisson regression model under multilevel frame work and the multinomial logistic regression for categorical characteristics. ET: embryo transfer; RR: risk ratio; IQR: interquartile range.

After adjusting for confounders, on average there was a 8% decreased risk of low birthweight among singletons born following blastocyst transfer versus those born following cleavage stage transfer (8.1% versus 9.0%; aRR 0.92; 95% CI 0.86, 0.99) (Table II); however, absolute risks were low in both groups. Blastocyst stage ET was associated with lower risk of being SGA in comparison to cleavage stage ET (8.0% versus 10.4%; aRR 0.83; 95% CI 0.78, 0.89). There was no statistically significant difference in the risk of very preterm birth or preterm birth, high birthweight, LGA, or healthy baby between the two groups. A total of 422 babies born with gestational age below 22 weeks and birthweight below 500 g were excluded as they were born outside the definition of ‘perinatal’, and 33 of these (7.8%) had a congenital anomaly. Since there may be a possibility of bias because of exclusion, a sensitivity analysis was carried out in which these babies were included in the analysis. We found that the results were consistent with our original findings.

**Table II:** Comparison of perinatal outcomes between singletons born following blastocyst and cleavage (reference) stage embryo transfer.

Perinatal outcomes	Live births following blastocyst stage ET (N = 28 814) n (%)	Live births following cleavage stage ET (N = 57 816) n (%)	Live births following blastocyst versus cleavage crude RR (95% CI)	Live births following blastocyst versus cleavage adjusted RR^@^ (95% CI)
Gestational age at birth				
Very preterm birth (vs full-term birth)	451 (1.6)	1000 (1.7)	0.91 (0.81, 1.01)	0.89 (0.77, 1.03)
Preterm birth (vs full-term birth)	2644 (9.2)	5232 (9.1)	1.01 (0.97, 1.06)	1.00 (0.94, 1.06)
Birthweight categories				
Low birthweight (vs normal birthweight)	2325 (8.1)	5220 (9.0)	0.88 (0.83, 0.92)	0.92 (0.86, 0.99)
High birthweight (vs normal birthweight)	2255 (7.8)	4915 (8.5)	0.90 (0.86, 0.95)	0.99 (0.93, 1.06)
Birthweight adjusted for gestational age	(n = 28 270)	(n = 56 777)		
Small for gestational age (vs appropriate for gestational age)	2249 (8.0)	5897 (10.4)	0.74 (0.71, 0.78)	0.83 (0.78, 0.89)
Large for gestational age (vs appropriate for gestational age)	3070 (10.9)	6158 (10.9)	0.97 (0.93, 1.02)	0.99 (0.93, 1.05)
Congenital anomaly	542 (1.9)	2398 (4.2)	0.46 (0.42, 0.50)	0.79 (0.71, 0.89)
Healthy baby	22 744 (78.9)	43 981 (76.1)	1.04 (1.03, 1.05)	1.00 (1.00, 1.02)

Very preterm birth (<32 weeks); preterm birth (<37 weeks); full-term birth (≥37 weeks); low birthweight (<2500 g); normal birthweight (2500–4000 g); and high birthweight (>4000 g). Healthy baby was defined as a baby born at or after 37 weeks of gestation, with birthweight between 2500 and 4000 g and no congenital anomalies. Small for gestational age (below 10th percentile birthweight adjusted for age); large for gestational age (above 90th percentile birthweight adjusted for age); and appropriate for gestational age (equal or above 10th percentile and equal or below 90th percentile birthweight adjusted for age).

@ Adjusted for age, female infertility characteristics such as tubal, ovulatory, male factor, unexplained, previous livebirths, and treatment characteristics such as type of treatment (IVF versus ICSI), number of eggs collected, and year of treatment. ET: embryo transfer; RR: risk ratio.

In the secondary analysis, we did not find any change in the association between stage of transfer and all perinatal outcomes between 2000–2008 and 2009–2017 in singleton analysis.

### Twin births

A total of 5194 twins were born following blastocyst transfer and 16 746 were born following cleavage stage transfer. Women in the blastocyst group were slightly older, had a higher proportion of previous livebirths, had more eggs retrieved and were more likely to have had a single ET compared to the cleavage stage group (Table III).

**Table III:** Comparison of baseline maternal and treatment characteristics between twin livebirths born following either blastocyst or cleavage stage embryo transfer.

MATERNAL/COUPLE CHARACTERISTICS	Live births following blastocyst stage ET (N = 5194) n (%)	Live births following cleavage stage ET (N = 16 746) n (%)	*P*-value
Maternal age at treatment (years)^@^	33.9 (3.91)	32.8 (3.86)	<0.001
Cause of infertility	860 (16.6)	3287 (19.6)	<0.001
Tubal disease			
Ovulatory disorder	775 (14.9)	2144 (12.8)	<0.001
Male factor	2133 (41.1)	7507 (44.8)	<0.001
Endometriosis	337 (6.5)	1148 (6.9)	0.359
Unexplained	1578 (30.4)	4481 (26.8)	<0.001
Duration of infertility (years)^@@^	4 (3, 6)	4 (3, 6)	0.402
Missing	3941	6154	
Previous live birth	608 (11.7)	1410 (8.41)	<0.001
Type of fertilization			<0.001
IVF	2171 (41.5)	8291 (49.5)	
ICSI	3023 (58.2)	8458 (50.5)	
Number of eggs collected	13 (10, 17)	10 (7, 14)	<0.001
Number of embryos transferred			<0.001
1	298 (5.7)	74 (0.4)	
2	4797 (92.4)	15 465 (92.3)	
3	99 (1.9)	1210 (7.2)	
Elective single ET			<0.001
Yes	233 (4.5)	25 (0.2)	
No	4961 (95.5)	16 724 (99.9)	

@ Mean and SD.

@@ Median (interquartile range).

The associations between baseline maternal and treatment characteristics and blastocyst (versus cleavage) stage embryo transferred were examined using a Poisson regression model under multilevel frame work and the multinomial logistic regression for categorical characteristics. ET: embryo transfer.

After adjustment for confounding factors, the risk of preterm birth (aRR 1.05; 95% CI 1.02, 1.10) was slightly higher among twins conceived following blastocyst transfer compared to those born following cleavage ET (Table IV). There was no statistically significant difference in the risk of very preterm birth, low birthweight, high birthweight, and congenital anomaly between the groups. However, the chance of healthy twins was lower for those born as a result of blastocyst compared to those born following cleavage stage transfer (aRR = 0.90; 95% CI 0.86, 0.95).

**Table IV:** Comparison of perinatal outcomes in twins between blastocyst and cleavage embryo transfer.

	Twin 1 (n = 21 943)		Twin 2 (n = 21 943)			
MATERNAL/COUPLE CHARACTERISTICS	Live births following b**lastocyst stage ET (N = 5194) n (%)**	Live births following c**leavage stage ET (N = 16** **749) n (%)**	Live births following **blastocyst stage ET (N = 5194) n (%)**	Live births following **cleavage stage ET (N = 16** **749) n (%)**	Crude RR 95% CI	Adjusted RR^@^ 95% CI
Gestational age at birth						
Very preterm birth (vs full-term birth)	470 (9.1)	1490 (8.9)	468 (9.0)	1496 (8.9)	1.12 (1.00, 1.26)	1.01 (0.89, 1.16)
Preterm birth (vs full-term birth)	2819 (54.3)	8259 (49.3)	2815 (54.2)	8257 (49.3)	1.10 (1.06, 1.15)	1.05 (1.02, 1.10)
Birthweight categories						
Low birthweight (vs normal birthweight)	2914 (56.1)	8907 (53.2)	3132 (60.3)	9676 (57.8)	1.12 (1.06, 1.18)	1.03 (0.97, 1.10)
High birthweight (vs normal birthweight)	13 (0.3)	43 (0.3)	14 (0.3)	42 (0.3)	1.09 (0.64, 1.84)	1.20 (0.65, 2.18)
Congenital anomaly	184 (3.5)	765 (4.6)	185 (3.6)	715 (4.3)	0.80 (0.69, 0.94)	1.06 (0.91, 1.25)
Healthy baby	1518 (29.4)	5798 (34.6)	1397 (26.9)	5295 (31.6)	0.85 (0.81, 0.89)	0.90 (0.86, 0.95)

Very preterm birth (<32 weeks); preterm birth (<37 weeks); full-term birth (≥37 weeks); low birthweight (<2500 g); normal birthweight (2500–4000 g); and high birthweight (>4000 g). Healthy baby was defined as a baby born at or after 37 weeks of gestation, with birthweight between 2500 and 4000 g and no congenital anomalies.

@ Age, female infertility characteristics such as tubal, ovulatory, male factor, unexplained, previous live births, and treatment characteristics such as type of treatment (IVF versus ICSI), number of eggs collected, and year of treatment. ET: embryo transfer; RR: risk ratio.

In the secondary analysis, the association between stage of transfer and congenital anomaly was different in the two time periods, 2000–2008 and 2009–2017 (interaction *P* < 0.001). In the time period 2000–2008, the risk of congenital anomaly was higher for blastocyst stage ET (aRR 1.59; 95% CI 1.32, 1.91) and between 2009 and 2017, the risk of congenital anomaly was marginally lower for blastocyst stage ET (aRR 0.68; 95% CI 0.50, 0.93). No significant interaction was found for the other outcomes (not shown).

### Singleton sibling pairs

**Figure 2.**
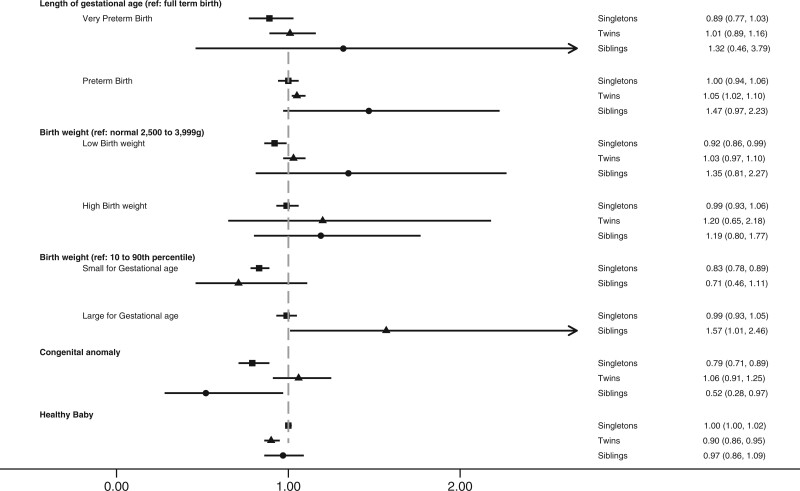
**Association between blastocyst versus cleavage stage embryo transfer and perinatal outcomes.** 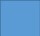 Singletons 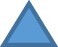 Twins 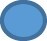 Siblings. Data are risk ratios (95% CI) (see also Table V).

Inclusion of the first two singleton siblings born following IVF treatment resulted in 5384 sibling pairs. Apart from maternal age and order of birth, other maternal characteristics (such as cause of infertility) were similar between the two comparison groups. Of the sibling pairs, 3158 (58.6%) were born following two cleavage stage ETs, 936 (17.3%) were born following two blastocyst ETs, 1028 (19.1%) were born following a cleavage ET for the first sibling and blastocyst ET for the second sibling, and 262 (4.9%) were born following blastocyst ET for the first sibling and cleavage stage ET for the second sibling (not shown). Only those singleton pairs (n = 1290) in which each sibling was born following a different ET strategy were included in the analysis. Singletons born following blastocyst transfer had a lower risk of congenital anomaly (2.8% versus 4.5%; aRR 0.52; 95% CI 0.28, 0.97) and a higher risk of being LGA (10.7% versus 9.8%; aRR 1.57; 95% CI 1.01, 2.46) compared to their siblings born following cleavage stage transfer (Fig. 2). There was no statistically significant difference in the risk of very preterm birth (1.2% versus 1.7%; aRR = 1.32; 95% CI 0.46, 3.79), preterm birth (8.2% versus 7.4%; aRR = 1.47; 95% CI 0.97, 2.23), low birthweight (6.4% versus 7.6%; aRR = 1.35; 95% CI 0.81, 2.27), and high birthweight (11.6% versus 8.3%; aRR = 1.19; 95% CI 0.80, 1.77) and being SGA (6.6% versus 9.6%; aRR = 0.71; 95% CI 0.46, 1.11) between singletons born following a blastocyst transfer and their siblings born following a cleavage stage transfer (Table V). There was not enough evidence from the data to suggest a statistically significant difference in the chance of having a healthy baby between siblings (76.1% versus 78.1%; aRR = 0.97; 95% CI 0.86, 1.09).

**Table V:** Comparison of perinatal outcomes between consecutive IVF singleton siblings born following blastocyst and cleavage embryo transfer.

*Perinatal outcome	Live births following blastocyst stage ET (N = 1290) n (%)	Live births following cleavage stage ET (N = 1290) n (%)	Live births following blastocyst versus cleavage transfer crude RR 95% CI	Live births following blastocyst versus cleavage transfer adjusted RR^@^ 95% CI
Gestational age at birth				
Very preterm birth (vs full-term birth)	16 (1.2)	22 (1.7)	0.76 (0.40, 1.46)	1.32 (0.46, 3.79)
Preterm birth (vs full-term birth)	106 (8.2)	96 (7.4)	1.10 (0.84, 1.46)	1.47 (0.97, 2.23)
Grouped birthweight categories				
Low birthweight (vs normal birthweight)	83 (6.4)	98 (7.6)	0.85 (0.61, 1.17)	1.35 (0.81, 2.27)
High birthweight (vs normal birthweight)	149 (11.6)	107 (8.3)	1.54 (1.15, 2.06)	1.19 (0.80, 1.77)
Birthweight adjusted for gestational age	(n = 1230)	(n = 1230)		
Small for gestational age (vs appropriate for gestational age)	81 (6.6)	118 (9.6)	0.65 (0.48, 0.89)	0.71 (0.46, 1.11)
Large for gestational age (vs appropriate for gestational age)	132 (10.7)	120 (9.8)	1.12 (0.84, 1.50)	1.57 (1.01, 2.46)
Congenital anomaly	36 (2.8)	58 (4.5)	0.62 (0.41, 0.94)	0.52 (0.28, 0.97)
Healthy baby	982 (76.1)	1008 (78.1)	0.97 (0.89, 1.06)	0.97 (0.86, 1.09)

Very preterm birth (<32 weeks); preterm birth (<37 weeks); full-term birth (≥37 weeks); low birthweight (<2500 g); normal birthweight (2500–4000 g); and high birthweight (>4000 g). Healthy baby was defined as a baby born at or after 37 weeks of gestation, with birthweight between 2500 and 4000 g and no congenital anomalies. Small for gestational age (below 10th percentile birthweight adjusted for age); large for gestational age (above 90th percentile birthweight adjusted for age), and appropriate for gestational age (equal or above 10th percentile and equal or below 90th percentile birthweight adjusted for age).

Fixed effect Poisson model was employed to estimate RR for congenital anomaly, healthy baby, and preterm birth and fixed effect multinomial logistic regression used to model birthweight and very preterm birth outcomes.

* This comparison examines the effect of change in ET on perinatal outcome from one IVF singleton sibling to the next.

@ Adjusted for age, order of birth, and treatment characteristics such as type of treatment (IVF versus ICSI), number of eggs collected, and year of treatment.

RR: risk ratio; ET: embryo transfer.

## Discussion

Our results show that singleton babies born following the transfer of a fresh blastocyst are at greater risk of being LGA but at lower risk of being born with a congenital anomaly than their siblings conceived from fresh cleavage stage embryos. Our sibling comparison removes much of the time-invariant residual confounding observed in earlier studies on this topic.

Singletons conceived following a blastocyst transfer were marginally less likely to be SGA than those born following a cleavage stage transfer. Singletons conceived following blastocyst transfer were less likely to be born with a congenital anomaly, which agrees with our singleton sibling finding. Twins conceived following a blastocyst transfer were marginally more likely to be preterm than those born following a cleavage stage transfer.

### Strengths of the study

A major strength of the study is the use of population-based national registry data over a 17-year period and inclusion of a complete birth cohort of singleton, twins and siblings. The capacity to link women with their IVF cycles allowed us to adjust for the clustering effect of multiple singletons born from the same women (Marconi *et al.*, 2019) and also to compare outcomes between siblings to disentangle the effects of the ET strategy itself from those related to maternal characteristics (Seggers *et al.*, 2016). By adjusting for order, the analysis accounts for differences in care between the first and second born sibling, for example mode of delivery.

### Limitations of the study

While we were able to adjust for a number of confounders, such as maternal age, cause of infertility, previous livebirths, number of eggs retrieved, type of insemination, and year of treatment, we were unable to adjust for BMI, ethnicity, race, smoking, and occurrence of vanishing twins as they are not reported in the HFEA dataset, as well as duration of infertility which was missing for more than 70% of women. As parity was not available in the registry data, previous livebirth status was used as a proxy in the adjusted model. As there has been a significant improvement in laboratory techniques and culture conditions during the 17-year study period, we have adjusted for the year of treatment.

As obstetric complications, such as pre-eclampsia and antepartum haemorrhage, are not recorded in the HFEA database, we were unable to include them in our analysis and it was also not possible to distinguish between spontaneous and iatrogenic preterm births.

Consent for research using IVF data changed from ‘presumed’ to ‘active opt in’ in October 2009. Thus, only data from patients who provided explicit consent for research were available in the linked HFEA database. Prior to 2009, 70–80% of linked patient records were available for research, but after 2009 this figure dropped to 40–50%.

Despite the use of appropriate statistical methods to mitigate against it, a degree of residual bias is inevitable in all observational studies and this is true for this study. Although our HFEA dataset allowed us to link cycles within each woman and undertake our sibling analysis to control for unmeasured maternal characteristics, there are still multiple limitations in our approach, not least because the ET strategy (blastocyst versus cleavage stage) was not allocated at random.

Though we were able to categorizes babies into SGA and LGA based on the UK centile chart of birthweight available for our singleton and siblings (Bonellie *et al.*, 2008), we could not do the same for twins (Briffa *et al.*, 2021) because the twin centile chart of birthweight for gestational age was stratified by chorionicity, which was not available in the HFEA dataset.

Frozen-thawed ET was not included in the study because the age of the ET was not available for frozen cycles in the dataset. With modern day IVF, the proportion of cycles adopting the freeze-all approach is increasing. The HFEA needs to take into consideration this limitation and capture the age of the frozen-thawed embryos when transferred for future research.

Our analysis of singleton siblings did not confirm the decreased risk of low birthweight but confirms the small decreased risk of congenital anomaly following blastocyst transfer found in singleton analysis. The precision of the aRR was low, because of the small number of events.

Finally, in the sibling analysis, while we were able to control for some factors that varied over time, such as maternal age, treatment type, and number of eggs collected, we were unable to control for other time varying factors. These could include maternal BMI, duration of subfertility and treatment-related factors, such as changes in IVF culture media over time and ovarian stimulation details. Such unmeasured time-varying factors may have resulted in residual confounding.

### Explanation of the findings

Exposing embryos to extended culture and blastocyst transfer appears to result in babies who are LGA (Mäkinen *et al.*, 2013). Though the actual mechanism is unclear, the literature suggests that embryo culture media could be influential in the genesis of the large offspring syndrome (Young *et al.*, 1998) in animals and LGA in humans (Dumoulin *et al*., 2010; Nelissen *et al.*, 2012). We also found a lower risk of congenital anomaly between a singleton born following blastocyst transfer and their singleton sibling born following cleavage stage transfer. However, it is worth noting that the precision around this finding is reduced by the smaller sample size available for the sibling analysis. The change in the risk of congenital anomaly between the time period 2000–2008 and 2009–2017 may be due to the availability of better techniques in the laboratory to improve the quality of embryos.

### Comparisons with other studies

#### Other studies in singleton siblings

To our knowledge, there are a limited number of studies that have compared perinatal outcomes between siblings (Kalra *et al.*, 2012; Luke *et al.*, 2017). However, they did not investigate the association between extended culture and outcomes such as congenital anomaly and LGA in siblings owing to limited sample size.

#### Other studies in singletons

Our study found a weak association between ET stage and low birthweight (aRR 0.92; 95% CI 0.86, 0.99) in singleton live births. However, the findings were not consistent with two similar studies (Kalra *et al.*, 2012; Chambers *et al.*, 2015). Kalra *et al.* (2012) showed limited evidence of an increase in risk of low birthweight for singletons born following extended embryo culture (aOR 1.23; 95% CI 0.99, 1.30). A population-based study of all ART cycles undertaken in Australia and New Zealand during 2009–2012 did not show an association between ET stage and low birthweight (aOR 1.00; 95% CI 0.92, 1.09) (Chambers *et al.*, 2015). Many other studies reported no association between embryo strategy and low birthweight (Dar *et al.*, 2014; Oron *et al.*, 2014; De Vos *et al*., 2015; Litzky *et al.*, 2018; Marconi *et al.*, 2019). Our finding of a lower risk of being SGA after fresh blastocyst transfer in singletons was consistent with a number of previous studies (Ishihara *et al.*, 2014; Zhu *et al.*, 2014; Ginström Ernstad *et al.*, 2016), as well as meta-analyses (Maheshwari *et al.*, 2013; Martins *et al.*, 2016; Wang *et al.*, 2017; Alviggi *et al.*, 2018).

In our study, the risk of congenital anomaly was lower in blastocyst stage ET compared with cleavage stage ET. This is in line with our findings in singleton siblings. In contrast to our findings, a Swedish register-based study, which partly adjusted for confounders such as maternal age, parity, smoking, BMI, and year of birth, reported a higher risk of congenital anomaly (aOR 1.43; 95% CI 1.14, 1.81) in infants born following blastocyst stage ET when compared to infants born following cleavage stage ET (Källén *et al.*, 2010). Other studies have found no evidence of an association between blastocyst versus cleavage-stage ET which may be due to limited sample size (Dar *et al*., 2013; Oron *et al.*, 2014, 2015; Ginström Ernstad *et al.*, 2016; Marconi *et al.*, 2019; Shi *et al.*, 2019).

#### Other studies in twins

Our finding of a small increased risk of preterm birth in twins born after blastocyst transfer is consistent with the US national level Society for Assisted Reproductive Technology database during 2004–2006 (aOR 1.39; 95% CI 1.29–1.50) (Kalra *et al.*, 2012). In contrast to this finding, data from Australia and New Zealand (Chambers *et al.*, 2015) showed that blastocyst transfer was associated with a lower odds of preterm birth among twins (aOR 0.80; 95% CI 0.70–0.93) born after blastocyst stage ET compared to cleavage stage ET. Both the studies (Kalra *et al.*, 2012; Chambers *et al.*, 2015) included additional potential confounders, such as number of prior assisted ART cycles, history of prior miscarriage, reduction in foetal heart rate on ultrasonography, and implantation rate, which were not available in the HFEA database.

### Implications for clinical practice and research

Our results provide some reassurance for the default position of extended culture as the absolute risks associated with this strategy are low.

The ideal option for generating unbiased data on perinatal outcomes is through follow-up studies of offspring born to women randomized to blastocyst or cleavage stage ETs. However, as such trials were conducted a while ago (Coskun *et al.*, 2000; Emiliani *et al.*, 2003; Papanikolaou *et al.*, 2005), follow-up is likely to be difficult owing to challenges associated with consent as well as logistics. New trials may not be feasible because of a lack of clinical equipoise.

Sibling studies are able to address the issue of confounding caused by unmeasured maternal factors but such analyses are not feasible on anonymized datasets, which are the norm for most national registries (Sunkara *et al.*, 2014; Marconi *et al.*, 2019). Further studies are required with singleton siblings in order to confirm the findings of birthweight, adjusted for sex and gestational age. Even where linking of cycles to women is possible, a number of factors which can influence outcomes, such as BMI, ethnicity, race, smoking, and duration of infertility, may not be recorded. Meta-analyses of published data on sibling outcomes are not possible owing to the very small number of studies reported (Kalra *et al.*, 2012; Luke *et al.*, 2017). Individual patient data meta-analysis of registry data across the world, which are able to provide a link between maternal and cycle level data, may overcome these shortcomings and provide an answer closer to the truth. However, such an endeavour will require collaboration, data governance and funding.

Our findings of lower risk of congenital anomaly is reassuring for couples seeking treatment for infertility, the physician and for IVF practice at the time when blastocyst transfer is being used widely across the sector. The lower risk of SGA is associated with a diminished risk of hospital admission for neonatal care and risk of chronic diseases in later life, including hypertension and cardiovascular diseases (Barker *et al.*, 2009). On the other hand, the perinatal risks of LGA include higher rates of caesarean delivery, postpartum haemorrhage, and neonatal shoulder dystocia and hypoglycaemia, as well as longer periods of hospitalization for newborn infants (Weissmann-Brenner *et al.*, 2012). LGA babies remain taller and heavier throughout childhood and have a high risk of developing adulthood obesity (Dietz, 1994; Parsons *et al.*, 1999).

## Conclusion

Our analysis of data from singleton siblings, partially corrected for maternal factors, suggests that babies conceived from blastocysts are at higher risk of being LGA but are less likely to have a congenital anomaly than those born after cleavage stage ET. However, the absolute risks of these outcomes are relatively low and there is insufficient evidence to challenge the practice of extended culture of embryos.

## Data Availability

The final dataset used in our analysis from this particular work is not available owing to HFEA strict privacy and confidentiality rules. The details of the original dataset can be found here: https://www.hfea.gov.uk/about-us/our-data/#ar, and may be requested by contacting the HFEA, register.research@hfea.gov.uk.
